# Application of Lab-on-Chip for Detection of Microbial Nucleic Acid in Food and Environment

**DOI:** 10.3389/fmicb.2021.765375

**Published:** 2021-11-04

**Authors:** Liu Yang, Wei Yi, Fangfang Sun, Mengjiao Xu, Zhan Zeng, Xiaoyue Bi, Jianping Dong, Yao Xie, Minghui Li

**Affiliations:** ^1^Department of Hepatology Division 2, Beijing Ditan Hospital, Capital Medical University, Beijing, China; ^2^Department of Gynecology and Obstetrics, Beijing Ditan Hospital, Capital Medical University, Beijing, China; ^3^Department of Hepatology Division 2, Peking University Ditan Teaching Hospital, Beijing, China; ^4^Department of Infectious Diseases, Haidian Hospital, Beijing Haidian Section of Peking University Third Hospital, Beijing, China

**Keywords:** food, environment, microorganism enrichment, LOC, isothermal amplification, biosensor

## Abstract

Various diseases caused by food-borne or environmental pathogenic microorganisms have been a persistent threat to public health and global economies. It is necessary to regularly detect microorganisms in food and environment to prevent infection of pathogenic microorganisms. However, most traditional detection methods are expensive, time-consuming, and unfeasible in practice in the absence of sophisticated instruments and trained operators. Point-of-care testing (POCT) can be used to detect microorganisms rapidly on site and greatly improve the efficiency of microbial detection. Lab-on-chip (LOC) is an emerging POCT technology with great potential by integrating most of the experimental steps carried out in the laboratory into a single monolithic device. This review will primarily focus on principles and techniques of LOC for detection of microbial nucleic acid in food and environment, including sample preparation, nucleic acid amplification and sample detection.

## Introduction

Pathogenic microorganisms refer to any microorganism capable of injuring its host by competing with it for metabolic resources, destroying its cells or tissues, or secreting toxins. The injurious microorganisms include viruses, bacteria, parasites, fungi, chlamydia, mycoplasma, etc. They can reside in food and the environment (e.g., water, soil, and air) and transmit disease, posing a serious threat to human health (Table 1). About 420,000 deaths and 600 million foodborne illnesses caused by 31 species of food-borne pathogenic microorganisms were reported in 2010. The burden of foodborne disease is rather high in low-income areas such as Africa, South-east Asia and the Eastern Mediterranean (Havelaar et al., 2015). Therefore, more convenient, rapid and economical microbial detection methods are needed to strengthen the detection of pathogenic microorganisms in food and environment, so as to achieve the purpose of prevention, timely diagnosis and isolation.

**TABLE 1:** Common pathogenic microorganisms in food, environment, related diseases, and main source.

Pathogen	Disease	Main source	References
*Norovirus*	Acute gastroenteritis.	Bivalve shellfish, vegetables, drinking water, surface water, sewage, recycled water, *etc*.	Kittigul et al., 2019; Miura et al., 2019; Sarmento et al., 2020
*Salmonella*	Salmonellosis, such as septicaemia, typhoid fever, acute gastroenteritis, *etc*.	Animal-derived foods such as pork, lamb, beef and poultry and poultry products such as laying hens, turkeys, eggs, drinking water, ocean, surface water, low temperature, organic manure improved clay, *etc*.	Arce et al., 2018; Phan-Thien et al., 2020; Mejia et al., 2021
*Campylobacter*	Acute self-limited enteritis, autoimmune diseases such as Miller Fisher syndrome, reactive arthritis, etc. Bacteremia and Guillain-Barre syndrome can occur in people with low immunity.	Poultry, especially broiler chickens, surface water, drinking water, *etc.*	Kaakoush et al., 2015; Ferrari et al., 2019; Sinulingga et al., 2020
*Escherichia coli*	Diarrhea, especially infants and childhood diarrhea in developing countries.	Dairy and meat products, surface waters, tap and well water, bottled drinking water, forest and pasture soils, *etc*.	Dusek et al., 2018; Abri et al., 2019; Topalcengiz and Danyluk, 2019; Vasconcellos et al., 2019
*Vibrio cholerae*	Choler, acute gastroenteritis, wound infection, otitis media, sepsis.	Fishes, shrimps, shellfish, crustaceans and other aquatic animals, coastal waters, reservoir, estuary, lake water,*etc*.	Fu et al., 2020; Daboul et al., 2020; Mavian et al., 2020
*Shigellai*	Bacillary dysentery.	Fresh vegetables, fresh fruit, meat, drinking water, surface water, municipal wastewater, *etc*.	Shahin et al., 2019; Vadde et al., 2019; Gebrewahd et al., 2020
*Hepatitis A virus*	Acute hepatitis A.	Bivalve shellfish, vegetables, fruit, *etc*.	Lowther et al., 2019
*Staphylococcus aureus*	Food poisoning.	Dairy products, meat, *etc*.	Velasco et al., 2018; Dai et al., 2019
*Rotavirus*	Diarrhea, especially in children below five years old worldwide.	Drinking water, surface water, sewage, recycled water and contaminated food, *etc.*	Miura et al., 2019; Tarek et al., 2019; Ahmed et al., 2020
*Clostridium tetani*	Tetanus.	Neutral or alkaline soils, with temperatures >20°C and humidity reaching at least 15%.	Popoff, 2020
*Bacillus anthracis*	Anthrax.	Clayey soils rich in organic matter and Ca^2+^, with pH above 6.0 and temperatures above 15.5°C.	Salgado et al., 2020
*Clostridium perfringens*	Gas gangrene, food poisoning.	Soil contaminated with feces, acid soils with pH values between 4.5 and 6.5.	Voidarou et al., 2011
*Yersinia pestis*	Plague.	Arid, highly saline soils.	Barbieri et al., 2020
*Leptospira*	Leptospirosis.	Warm, moist soils. There is a significant positive relationship between presence of Leptospira and concentration of iron, manganese and copper in soil.	Lall et al., 2018; Cucchi et al., 2019
*Soil-transmitted helminths*	Diarrhea, malnutrition, anemia, stunted growth, and impaired intellectual development.	Warm moist soil contaminated with feces.	Moser et al., 2019
*Mycobacterium tuberculosis*	Tuberculosis, such as pulmonary TB, TB meningitis, and TB lymph nodes.	Bioaerosol and droplets containing *Mycobacterium tuberculosis* exhaled by patients with active tuberculosis.	Patterson et al., 2020
*Measles virus*	Measles.	Bio aerosol and droplets containing measles virus, produced when an infected person coughs.	Zemouri et al., 2020
*Legionella*	Legionnaires’ disease.	Aerosols containing Legionella bacteria from cooling tower or other air-conditioning systems.	Crook et al., 2020
*Varicella-zoster virus*	Varicella, zoster.	Aerosol containing varicella-zoster virus from blistering skin lesions.	Lachiewicz and Srinivas, 2019
H1N1 virus	Bird flu.	Biological aerosols and droplets containing influenza A viruses produced when an infected person coughs, speaks, and sneezes.	Prost et al., 2019
COVID-19	Novel coronavirus pneumonia.	Respiratory droplets and air pollution particles (>1 μm)	Nazarenko, 2020
Non-tuberculous mycobacteria	Opportunistic lung infection.	It is ubiquitous and abundant in soil, but most of it is abundant in cold, damp, acidic soil, and the abundance is positively correlated with the content of iron in soil.	Walsh et al., 2019; Glickman et al., 2020
*Sporothrix*	Sporotrichosis.	Its distribution is related to a variety of plants, flowers, sawdust, reed leaves, corn stalks, leaves and sawdust, etc., and can survive in soil at temperatures from 6.6 to 28.84°C and 37.5% to 99.06% relative humidity.	Ramírez-Soto et al., 2018
*Aspergillus fumigatus*	It can lead to invasive lung disease in immunocompromised patients.	Can live in a wide range of pH and moisture conditions in the soil, heat resistant but not thermophilic, continuous temperature of 60°C will cause significant growth slowdown.	Wang D. et al., 2018; Stewart et al., 2020

Cell culturing and identification of the pathogen type by microscopy or other biochemical tests is the most common method of detecting pathogenic microorganisms, but it takes several days. In addition, some bacteria such as *Staphylococcus aureus* and *E. coli* can enter a viable but non-culturable state under severe harsh survival pressure. In this case, these bacteria remain to be harmful because of their virulence and pathogenicity (Chen et al., 2020; Liao et al., 2021). Thus, scientists have designed nucleic acid-based or immunological detection methods such as DNA probe, polymerase chain reaction (PCR), reverse transcription polymerase chain reaction (RT-PCR), lateral flow dipstick (LFD), enzyme linked immunosorbent assay (ELISA) and enzyme linked fluorescent assay (ELFA), etc. Unfortunately, most of them are expensive and unaffordable in low-income areas. What’s more, these technologies usually rely on sophisticated instruments and trained operators, which prevents them from on-site testing of food and environmental pathogenic microorganisms.

Point-of-care testing (POCT) is carried out in the field of sampling. It can quickly obtain results by using portable analytical instruments, while no professional laboratorians are needed. The development of POCT equipment that can be applied to low-income developing countries should follow the “ASSURED” principles as proposed by the World Health Organization, namely “(1) affordable, (2) sensitive, (3) specific, (4) user-friendly, (5) rapid and robust, (6) equipment-free and (7) deliverable to end-users” (Chen et al., 2019). Currently a research hotspot in the field of POCT is microfluidic technology, which allows precise control of micro-scale fluids in micro-nano scale space. Microfluidic technology offers unique advantages such as faster response, smaller volume of sample and reagent, greater sensitivity, shorter diffusion distances, and smaller system sizes. Lab-on-chip (LOC) uses microfluidic technology to integrate all steps from sample preparation to sample testing into a micro device. Here, we reviewed the recent advances in the application of LOC in food and environmental microbial detection. Since nucleic acid detection techniques have made great progress recently, this paper focuses on application of LOC for the nucleic acid detection of food and environmental microorganisms.

## Overview of Lab-On-Chip

Lab-on-chip, also known as a micro total analysis system, uses microfluidic technology to integrate sample input, dilution, reaction and separation within a single monolithic device. The micro total analytical system was first proposed by A. Maz, N. Gaber and H.M. Idmer in 1990. Based on flow injection analysis, chromatography and electrophoresis, the micro total analytical system can achieve faster and more efficient chromatographic separation, faster electrophoresis separation speed, shorter transmission time, and remarkably reduce the consumption of carrier, reagent or mobile phase (A. Manz et al., 1990). Since then, LOC has attracted attention of researchers in biology, medicine, chemistry, electronics, materials science and many other fields. The preparation materials were developed from silicon and quartz glass to high polymer such as thiol-ene polymer, polystyrene, polycarbonate, polydimethylsiloxane (PDMS), polymethylmethacrylate (PMMA), cellulose acetate paper (Mauk et al., 2015; Hansen et al., 2018; Xiong et al., 2019; Hasan et al., 2020; Yin et al., 2020). The chip was prepared with ultraviolet lithography, soft lithography (Hansen et al., 2018), and 3D printing technology (Kim et al., 2018). New materials and fabrication processes make it possible to produce large numbers of identical chips within a short time.

Most of the existing LOC equipment for microbial detection in food and the environment are based on immunological principles, as immunological-based methods are simple to operate. In contrast, nucleic acid testing often requires elaborate sample handling to release, separate, and concentrate nucleic acid, and is not commonly used in LOC equipment. But sensitivity and specificity of nucleic acid testing is superior to immune assays. By *in vitro* amplification, nucleic acid-based tests can achieve a higher specificity and sensitivity (1,000 times or more) than immunoassay (Mauk et al., 2015).

**FIGURE 1:**
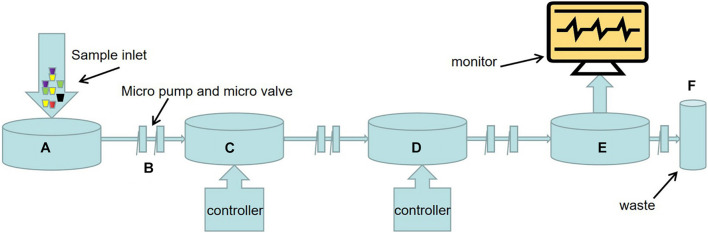
Schematic diagram of LOC process for nucleic acid detection of food and environmental microorganisms. **(A)** Sample injection. **(B)** There are a large number of micro-pumps and micro-valves on the chip to precisely control the flow direction and flow rate of microfluids. **(C)** Sample preparation including pathogen capture, cell lysis, nucleic acid extraction and purification, etc. The methods of pathogen capture mainly include microsphere, filter or membrane, dielectrophoresis, magnetophoresis, acoustophoresis, etc. This part often needs external electric field, magnetic field, ultrasonic, and its strength is controlled by the controller. **(D)** Nucleic acid amplification. In addition to traditional PCR, various emerging isothermal nucleic acid amplification techniques have been applied to LOC equipment. The substrate used for nucleic acid amplification is packed into a micro chamber in advance, and the temperature of the reaction process is controlled by a temperature controller. **(E)** Sample detection devices, primarily sensors. Such as fluorescence sensors, surface plasma resonance (SPR) sensors, surface enhanced Raman scattering (SERS) sensors, electrochemical biosensors. **(F)** Results are visually displayed on the display.

As shown in Figure 1, the main steps of nucleic acid detection of microorganisms in the food environment include pathogen capture, cell lysis, nucleic acid extraction and purification, nucleic acid amplification and nucleic acid detection. Each step can be accurately controlled by micro-pump, micro-valve, and micro-column on the chip (Romao et al., 2017).

## Sample Preparation in LOC for Nucleic Acid Detection of Food and Environmental Microorganisms

Generally the content of target microorganisms and nucleic acids in the matrix of food environmental samples is too few to be used directly for nucleic acid amplification. Moreover, the efficiency of nucleic acid amplification reaction is affected by many disadvantages in the complex sample matrix, such as plasmin and calcium ion in milk, myoglobin in muscle, humic acid in soil, particulate matter in indoor air, etc. (Hedman and Rådström, 2013). Therefore, a reliable sample preparation method, namely pathogen capture, is required to isolate the target microorganisms from the complex sample matrix before nucleic acid amplification. After being isolated from a complex sample matrix, the target microorganism can be used for nucleic acid amplification after elution, cell lysis, nucleic acid extraction and purification. Currently, methods of pathogen capture commonly used in LOC devices include microsphere, filter or membrane, dielectrophoresis (DEP), magnetophoresis, acoustophoresis, etc, which are highlighted below.

### Microspheres, Filters or Membranes

The target microbes in samples can be attracted by specific antibodies in the microspheres, and then detected quickly by immunofluorescence and other technologies (Chen and Park, 2018). Zhao et al. (2021) developed a highly sensitive fluorescent immunosensor to detect *E. coli O157:H7* in milk. In this system, the target cells are captured by the microspheres marked with carbon dots (CDs). CDs has excellent optical properties, so the microspheres have strong fluorescence intensity, good stability and uniformity, and great potential as a fast and sensitive tool for detecting pathogens in milk and other foods. Cai et al. (2019) modified magnetic nanoparticles with anti-*Salmonella* monoclonal antibodies for the enrichment of *Salmonella* Typhimurium from samples. Then they were conjugated with anti-*Salmonella* polyclonal antibody and catalase modified polystyrene microspheres to form magnetic nanoparticle-bacteria-polystyrene-catalase sandwiches. Catalase from the complex passes through a micromixer to catalyze the decomposition of hydrogen peroxide to produce oxygen. The oxygen increases the pressure in the microchannel and pushes the indicative red dye solution to move along the channel. The movement distance of the red dye can be visually seen using calibration scales and is related to the number of *Salmonella* typhimurium. In the LOC equipment based on nucleic acid detection, various methods such as magnetophoresis and acoustophoresis are needed to separate the bacteria-microspheres complex from the microspheres without bacteria. After washing and cell lysis, DNA/RNA is obtained for nucleic acid amplification (Kant et al., 2018). Kubo et al. (2020) designed a LOC device to detect *Salmonella* in egg yolks using magnetic beads modified with anti-*Salmonella* antibodies. After thermal lysis, the target genes were amplified by PCR, and then detected by fluorescence probe.

Filters or membranes can also be used for pathogen enrichment. Kim and Oh (2019) inoculated *E. coli O157:H*7 on beef, then filtered the beef homogenate at a concentration of 10^2^CFU/mL with 0.45 μm cellulose fiber membrane. DNA in concentrated E. coli was amplified and analyzed by loop-mediated isothermal amplification (LAMP). The results showed that the sensitivity of sample testing of the filtered samples was 100 times higher than that of the unfiltered samples, for the homogenate at a concentration of 10^2^CFU/ml, while the total reaction time from sample preparation to confirmation of *E. coli* was within 3 h. Vibbert et al. (2015) used hollow fiber membranes with a diameter of 0.28 mm to microfilter the chicken homogenate after endopeptidase treatment, which could increase the microbial concentration to the detectable level within a few of hours.

### Dielectrophoresis

Dielectrophoresis is a method of separating suspended particles by producing a polarizing force in a non-uniform electric field. The particles in DEP are uncharged but must be polarizable (Zhang et al., 2019). Large amounts of electrical charges can be generated at the surface of the polarizable particles exposed to a non-uniform electric field. These charges form dipoles (a pair of charges with opposite signs that are very close together) arranged in parallel with the applied magnetic field. Each half of the dipole in a non-uniform electric field is subjected to an unequal force, so the net force on the particle is not zero, which then pushes the particle toward or away from a region of strong electric field. Cells are typically polarizable particles. The net force depends on the dielectric constant, size and shape of the particle, and the dielectric constant of the medium, so it can selectively target the particle according to its phenotype (Khoshmanesh et al., 2011). Abdullah et al. (2019) used positive dielectrophoresis (pDEP) based focusing electrodes and biosensors to detect *Salmonella* in concentrations as low as 10 pieces/mL from chicken products in less than 1 h. Cai et al. (2018) designed a kind of microfluidic device based on pDEP, which integrated H-type filter desalination and pEDP, and could directly enrich *E. coli* from physiological samples with high conductivity and viscosity such as milk. In the main channel of the H-type filter, the electrolyte is continuously diffused into the deionized water, while the bacteria remain in the sample. After desalination, the sample is pumped into the DEP chamber, where the bacterial cells are captured through the pDEP. Jasim et al. (2019) designed a microfluid-based impedance biosensor that could rapidly detect three *Salmonella* serogroups simultaneously. It consists of three microchannels, and in which *Salmonella* cells are focused on the centerline and guided to the sensing area by pDEP to obtain highly concentrated samples.

### Magnetophoresis

Magnetophoresis is a technology that uses a flexible and controllable magnetic field to manipulate the motion of magnetic beads in a microchannel (Cao et al., 2014). The magnetic beads can be separated according to their different size and magnetic content, which leads to different direction of magnetic beads deviating from laminar flow (Pamme and Manz, 2004). Magnetophoresis can be used to separate various bacterial microbead complexes from microbeads without bacteria. Ngamsom et al. (2016a) used two different commercial magnetic beads (Dynabeads^®^ *Salmonella* resistant magnetic beads and Hyglos-Streptavidin magnetic beads) to multiple separate *Salmonella* typhimurium and *E. coli* in food preconcentration. The mixed cultures of the bacterial microbead complexes are introduced into the separation chamber with the buffer, and the two types of microbeads are isolated because of different magnetic forces under the influence of the array magnets. Malec et al. (2018) designed a microfluidic device based on magnetophoresis and obtained reliable parameters for predicting *E. coli* concentration. In this system, *E. coli* is captured by streptavidin coated magnetic particles (MPs) to form magnetically labeled bacteria (MLBs). The MLBs are suspended in the liquid of the microchannel and are accelerated toward the exit by means of a magnetic field gradient. The magnetic field gradient is generated by the integrated microconductor and controlled by the microcontroller. As a reference, the reference MPs was added to the same liquid in parallel microchannel, and the velocities of MLBs and reference MPs were compared in real time using a digital camera mounted on an optical microscope combined with particle track tracking software.

### Acoustophoresis

Acoustophoresis can be used to separate particles with different acoustic physical properties without labeling, that is, antibody staining or other labeling is not needed. Acoustophoresis uses ultrasonic waves in microchannels to control the migration of suspended particles of different size, compressibility, and density. The denser or less compressible particles move more rapidly toward the pressure node in the center of the channel than the less dense or more compressible particles. Therefore, acoustophoresis can selectively direct suspended particles with different acoustic physical properties to different microchannel outlets (Olm et al., 2021). The recovery of *Salmonella* typhimurium from chicken and beef samples by acoustophoresis has achieved high recovery rates (60–90%) (Ngamsom et al., 2016b). GN6 aptamer is a type of aptamer that binds specifically to gram-negative bacteria, so when mixed with a complex sample matrix, it can selectively capture gram-negative bacteria and leave gram-positive bacteria behind. Lee S. et al. (2019) mixed microspheres coated with GN6 aptamers with samples and injected them into microchannels. As the mixture enters the acoustic standing wave field, the microspheres bound to gram-negative bacteria migrate along the buffer center and exit the system through the outlet center. Gram-positive bacteria remained in the original buffer flow along the side wall and were removed through the side wall. Using ultrasound to manipulate particles in microfluidics channels is a promising research direction. Aghakhani et al. (2021) reported an acoustophoresis system based on flexural wave, which can capture micron-sized particles or cells on the soft wall. The acoustophoresis system is expected to play an important role in enhancing immunoassays and particle sensors.

## Nucleic Acid Amplification in LOC for Nucleic Acid Detection of Food and Environmental Microorganisms

### Polymerase Chain Reaction

Polymerase chain reaction (PCR) is a molecular biology technique to greatly increase the amount of nucleic acid *in vitro* (Saiki et al., 1988). After the completion of nucleic acid amplification, methods such as gel electrophoresis are needed to quantify the DNA. Real-time quantitative PCR is a technique for real-time amplification of target DNA and quantification of products in a system, but it’s not absolutely quantitative (Singh and Roy-Chowdhuri, 2016). Digital PCR is a kind of absolute quantitative method. It mainly disperses the diluted nucleic acid solution into microreactors or droplets of the chip, and the number of nucleic acid templates in each reactor or droplet is ≤1. After the PCR cycle, only the reactor with a template of the nucleic acid molecule will give a fluorescence signal (Catarsi, 2019). Reverse transcription polymerase chain reaction (RT-PCR) is used to detect RNA. In RT-PCR, RNA is firstly transcribed into complementary DNA (cDNA), which can then be amplified by PCR (Elfman and Li, 2020). Microfluidic digital PCR has been successfully used to detect norovirus and hepatitis A in soft berries, water samples and lettuce, and hepatitis E virus in naturally contaminated pig liver products (Coudray-Meunier et al., 2015; Martin-Latil et al., 2016; Fraisse et al., 2017). A shunt PCR method was reported by Salman et al. (2020). In this microfluidic device, the PCR reaction chamber can be heated or cooled in a short period of time by a thin layer of fluid and a large static heating system with high thermal inertia elements and stabilized at a preset reaction temperature. The reaction chamber is not fixed, but is cycled between the reaction temperatures required for the denaturation, annealing and extension stages. Fluorescent dyes were embedded in the PCR products, and the whole PCR process was monitored in real time by the fluorescence detection lock-on photodetector.

### Isothermal Nucleic Acid Amplification

Polymerase chain reaction-based nucleic acid amplification is complex and requires expensive instruments, which does not meet the goals of LOC development. Isothermal nucleic acid amplification is an ideal alternative to PCR given that it is performed at constant temperatures and can be used for nucleic acid amplification without programmable thermal cyclers. In recent years, isothermal nucleic acid amplification technology has been developed rapidly, and the most mature ones include loop-mediated isothermal amplification (LAMP), recombinase polymerase amplification (RPA), helicase-dependent amplification (HDA), rolling circle amplification (RCA), nucleic acid sequence-based amplification (NASBA), cross priming amplification (CPA) (Woźniakowski et al., 2018), strand swat amplification (SDA) (Walker et al., 1994) and recombinant enzyme assisted amplification assay (RAA) (Shen et al., 2019).

#### Loop-Mediated Isothermal Amplification

**FIGURE 2:**
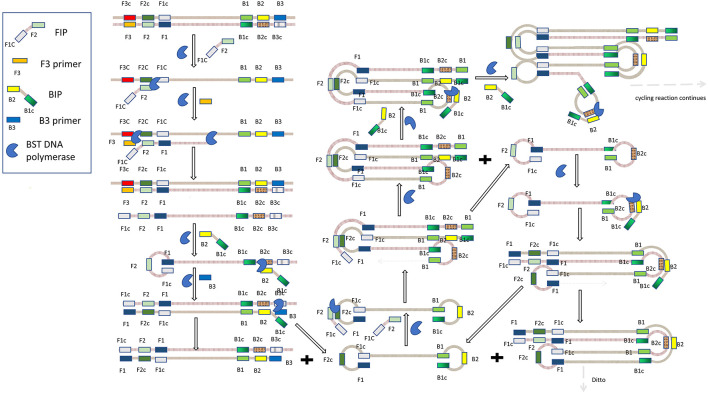
Schematic diagram of loop-mediated isothermal amplification.

Loop-mediated isothermal amplification, which was first proposed by Notomi et al. (2000), has the advantages of high specificity, sensitivity, speediness and simplicity (Guan and Ma, 2014). As shown in Figure 2, it requires a BST DNA polymerase with strand displacement activity and four primers specifically designed to identify six different regions of the target DNA, and the reaction takes place at 60–65°C. The four primers include forward internal primer (FIP), F3 primer, reverse internal primer (BIP), B3 primer. As is shown in Figure 2, FIP consists of F2 and F1c region, which are complementary to F2c and F1 regions in the two strands of DNA, respectively. The primer of this structure is the basis of the formation of stem-loops in the subsequent amplification process. The F3 primer, also known as forward outer primer, is complementary to the F3c region. Similarly, BIP consists of B2 and B1c region, and B3 primer, also known as backward outer primer, is complementary to B3c region. At first, the F2 region of FIP hybridizes with the F2c region of DNA to initiate complementary strand synthesis with the action of BST DNA polymerase. The F3c region is exposed at this moment. However, since F3 primer is shorter than FIP and has a lower concentration in the system, F3 primer will hybridize with F3c region for a period of time after FIP mediates amplification with the action of BST DNA polymerase and releases complementary chain connected to FIP. The end of the released complementary chain includes F1 and F1c regions, thus forming a loop, while the other end serves as a template for BIP-initiated DNA synthesis and subsequent B3 prime-initiated strand replacement. The first stage eventually produces dumbbell-shaped DNA, which serves as the starting material for the second stage of the LAMP reaction. The LAMP reaction then continues in this manner, eventually producing a mixture of stem-loop DNA with different stem lengths and cauliflower shaped structures with multiple loops (Notomi et al., 2000; Li et al., 2016).

Zhou et al. (2021) developed a microfluidic chip integrating real-time fluorescence and LAMP technology, which could simultaneously detect 10 pathogenic microorganisms. They used a universal genomic DNA extraction kit in advance to extract total DNA from the sample and then added it to the system for sample testing. The average detection time was less than 30 min. The limits of detection of bacterial genomic DNA was 10^0^–10^–1^ pg/μL, and the limits of detection of recombinant plasmid DNA was 10^–4^–10^–5^ pg/μL. Compared to the conventional microbial detection method, its specificity and sensitivity were 85.53% and 93.52%, respectively. Trinh and Lee (2018) designed a LAMP-based plastic microdevice for the detection of *E. coli O157:H7*, *Staphylococcus aureus*, *Salmonella*, and others in milk samples. Jiang et al. (2016) developed a LAMP-based microfluidic chip for rapid capture, enrichment and detection of airborne *Staphylococcus aureus*. The entire analysis process took about 4 h and 40 min, and the detection limit was as low as about 27 cells. LAMP has also been used in LOC applications for *Vibrio parahaemolyticus* in aquatic products, *Aspergillus fumigata* in clinical and environmental isolates, and *Salmonella* in food (Pang et al., 2017; Wang et al., 2020; Yu L. S. et al., 2020).

#### Recombinant Enzyme Polymerase Amplification

Recombinant enzyme polymerase amplification was first proposed by Piepenburg et al. (2006). The whole reaction system consists of bacteriophage recombinase uvsX and cofactor uvsY, primers, single-stranded DNA-binding protein (SSB) T4 GP32, DNA polymerase, deoxynucleotide triphosphate (dNTP) and buffer, etc. RPA is usually performed under isothermal conditions between 37°C and 42°C, which is suitable for on-site pathogenic diagnosis in field lack of instruments and for preventing heat-induced DNA mutations. The reaction time is usually 15–40 min, which is shorter than most PCR reactions (Yang et al., 2018; Lee J. et al., 2019).

**FIGURE 3:**
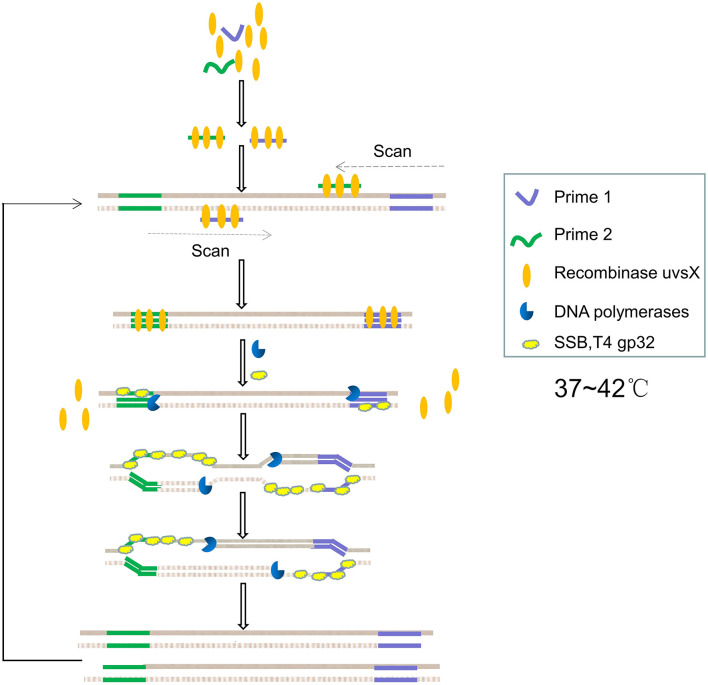
Schematic diagram of recombinant enzyme polymerase amplification.

The principle of PRA is shown in Figure 3. Firstly, the recombinase forms a complex with the primers, which scans the DNA sequence and inserts the primers into homologous locus through the strand displacement activity of the recombinant enzyme. Meanwhile, SSB stabilizes the replaced single-stranded DNA. The recombinase then disintegrates, making it easy for DNA polymerase with strand displacement activity to enter the 3′-end of the primers to prolong the primers. Exponential amplification is achieved by repeating the process over and over again (Lobato and O’Sullivan, 2018). Lee et al. (2021) introduced a high-performance nanogap impedimetric sensor that uses RPA to amplify nucleic acids in real time. The nanogap impedimetric sensor was immersed in the RPA reaction solution to detect *E. coli O157:H7*. The amplification of the target DNA was evaluated by impedance spectroscopy changes per minute during the RPA process. Yin et al. (2020) presented an integrated multiplex digital RPA (ImdRPA) microfluidic chip that successfully detected *E. coli O157:H7*, *Salmonella* enteritis and *Listeria* monocytogenes in milk within 45 min. Ahn et al. (2018) developed an RPA method based on a paper chip device, which is made by simply stacking functional paper and drying the RPA reagent and fluorescent probe in the reaction zone of a poly (ether sulfone) membrane. *E. coli, Staphylococcus aureus* and *Salmonella typhimurium* can be detected simultaneously based on fluorescent signal of paper chip, and the detection limit was 10^2^CFU/mL.

#### Helicase-Dependent Amplification

Helicase-dependent amplification was first proposed in 2004 by Vincent et al. (2004). The whole reaction system is composed of helicase, SSB, DNA polymerase, two primers, dNTP, and buffer. The helicase and the polymerase must work jointly to prevent the polymerase from being replaced by the helicase. Pairs of helicases/polymerases that work together in natural systems must therefore be used. Vincent et al. initially used *E. coli* UvrD helicase/DNA polymerase I Klenow fragment, which can be performed at 37°C. If thermophilic helicase/polymerase is used for HDA, the reaction can be carried out at 60∼65°C, which is called thermophilic helicase dependent HDA (tHDA) (Xu et al., 2018).

**FIGURE 4:**
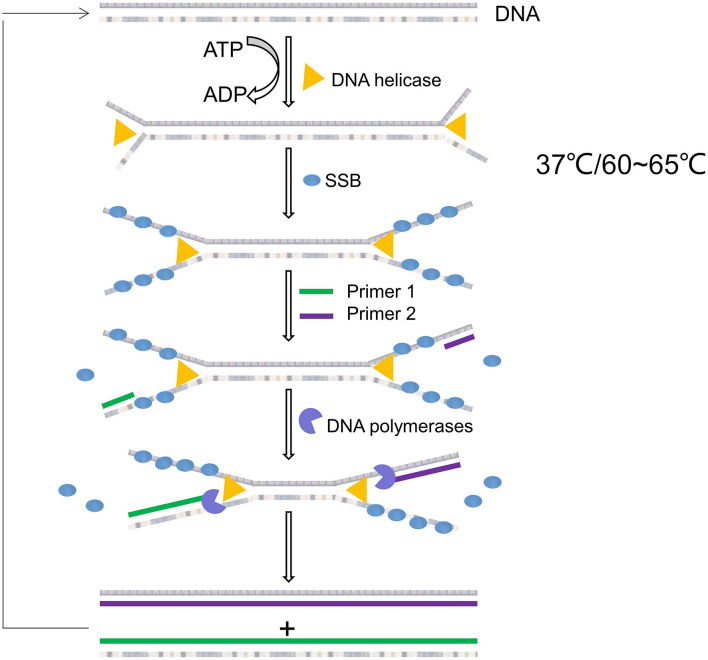
Schematic diagram of helicase-dependent amplification.

The principle of HDA is shown in Figure 4. In the reaction system, the helicase uses energy of adenosine triphosphate hydrolysis to break the hydrogen bonds between the complementary bases of double-stranded DNA, thus untying the double-stranded DNA. Therefore, the dNTPs mixture must be rich in dATP as a cofactor of the helicase. If *E. coli* UvrD helicase is used, the methyl directed mismatch repair protein (MUTL protein) should be added to the reaction system. This collaboration between UvrD helicase and MutL protein is associated with the repair of DNA mismatches in *E. coli*. After the double chain is unchained, the SSB binds to the unchained single chain to prevent recombination of the complementary chains. After stabilizing the DNA, the two primers bind to the target sequence, and DNA polymerase extends the primers using dNTP to produce a double-stranded amplification product (Barreda-García et al., 2018). Chen et al. (2015) extracted genomic DNA from lysed bacteria using silica coated magnetic nanoparticles and amplified it using tHDA to detect Staphylococcus aureus in dairy and meat products. The detection limit of the system was 5 × 10^0^ CFU/mL for milk powder samples and 5 × 10^1^ CFU/mL for pork samples within less than 2 h. HDA has also been used to detect *Enterobacte*r sakazakii in infant formula with high sensitivity (94%) and specificity (100%) (Xu et al., 2018).

#### Rolling Loop Amplification

**FIGURE 5:**
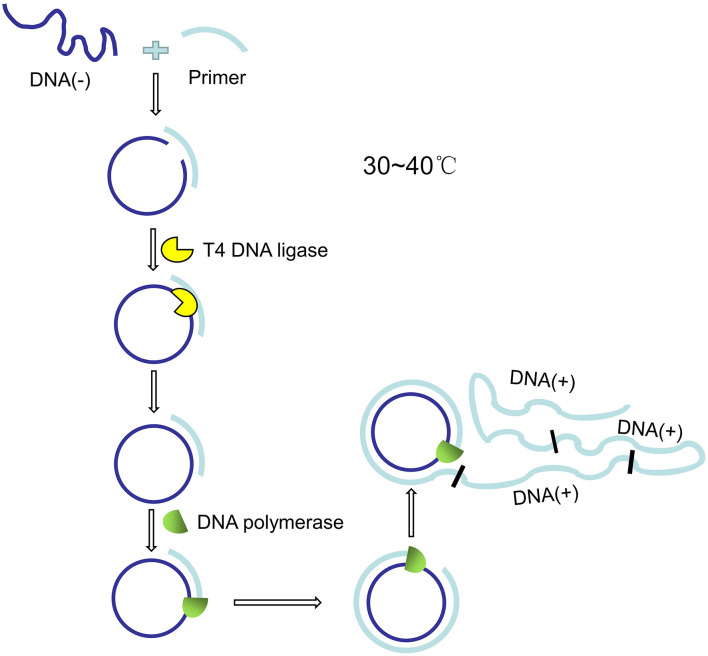
Schematic diagram of rolling loop amplification.

The concept of RCA was first presented in the 1990s (Liu et al., 1996), using circular DNA/RNA templates and special DNA or RNA polymerases to amplify short DNA or RNA primers into long single-stranded DNA or RNA. A typical DNA amplification reaction system consists of primers P, T4 ligase, bacteriophage φ29 DNA polymerase, dNTP and buffer, and the reaction usually takes place at 30∼40°C. The principle of RCA is shown in Figure 5.

The circular DNA/RNA template is generated by hybridizing primer P with long chain T. Primer P is designed to have a larger and a shorter overlap with T, and the long chain T can be converted into circular molecules by T4 ligase. The RCA reaction for DNA amplification is performed with bacteriophage φ29 DNA polymerase, which has special chain displacement properties. The polymerase begins to synthesize the complementary chain at primer P. After a round of polymerization, it displaces the newly synthesized strand and continues to polymerize, which eventually leads to the formation of a single long strand with a repeating sequence of T (Beyer et al., 2005). Minero et al. (2019) designed a microfluidic device that uses magnetic microspheres to capture the target microorganism and amplify nucleic acids with RCA, which is expected to be used in the detection of microorganisms in food and environment. Jiang et al. (2020) proposed a novel dual-RCA microfluidic platform for the detection of *E. coli O157:H7*, which could significantly improve the detection signal by about 250 times. RCA was used twice in this process. The first RCA was used for *in situ* amplification of aptamers conjugated to the surface of microchannels. The aptamers are used to capture *E. coli O157:H7*, and its nucleic acid sequence will be amplified several times by RCA. The target cells captured by RCA amplified microchannels were 3 times more than those without RCA amplified microchannels. The second RCA reaction was aimed to amplify the detection signal of *E. coli O157:H7*. The product was a long extended repetitive *E. coli O157:H7* DNA sequence complementary to the signal probe. The microfluidic platform can be used in a variety of food matrices, including orange juice and milk, with a detection limit of 80 cells/mL.

#### Nucleic Acid Sequence-Dependent Amplification

**FIGURE 6:**
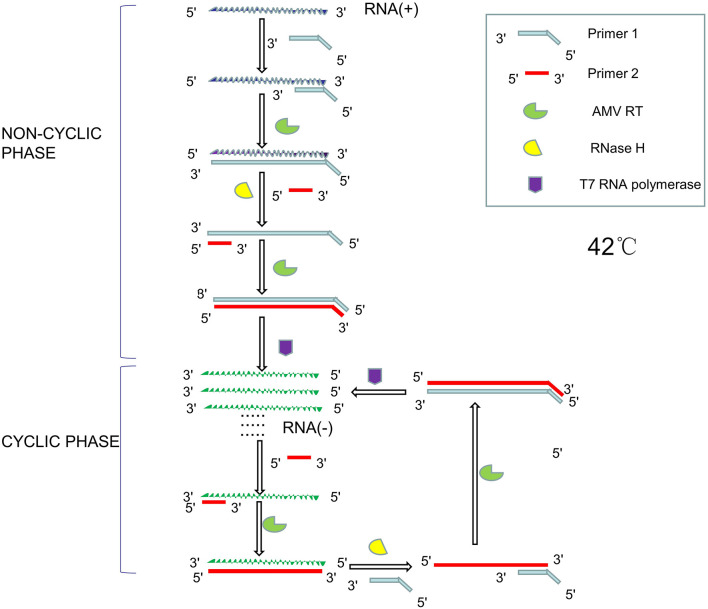
Schematic diagram of NASBA principle.

Nucleic acid sequence-dependent amplification is a technique for isothermal RNA amplification firstly proposed by Compton (1991). The entire reaction system consist of Avian Myeloblastosis Virus Reverse Transcriptase (AMV RT), bacteriophage T7 RNA polymerase, ribonuclease H, two primers (the 5′ end of primer 1 contains a T7 promoter sequence recognized by bacteriophage T7 RNA polymerase), dNTP, nucleotide triphosphates (NTP), and buffer. AMV RT can synthesize DNA using either DNA or RNA as templates. Bacteriophage T7 RNA polymerase is a DNA-dependent 5′→3′RNA polymerase that highly specifically recognizes the T7 promoter sequence. RNase H specifically hydrolyzes RNA in the DNA-RNA heterozygote. The reaction usually takes place at around 42°C (Huang et al., 2019). Its principle is shown in Figure 6.

The reaction consists of two parts, namely non-cyclic phase and cyclic phase. In the non-cyclic phase, the forward primer (primer 1) hybridizes with the single-stranded RNA to synthesize complementary DNA strands with the action of AMV RT, and then RNase H hydrolyze the RNA strand. The reverse primer (primer 2) hybridizes with the remaining DNA single strand, and the DNA is synthesized to form double stranded DNA with the help of the AMV RT. The T7 RNA polymerase then recognizes the promoter sequence of the DNA and transcribes it into single stranded RNA. In the cyclic phase, single-stranded RNA binds to reverse primers to synthesize single-stranded DNA under the action of AMV RT. The RNA strand in DNA-RNA heterozygote strand will be hydrolyzed by RNase H, and the remaining DNA is hybridized with forward primers to synthesize the DNA sequence with the T7 promoter sequence with the help of AMV RT. T7 RNA polymerase can then use DNA as a template to produce large amounts of single-stranded RNA (Wang J. et al., 2018). Aufdembrink et al. (2020) used a set of fluorescent aptamers as NASBA labels to significantly improve sensitivity of the assay, which can be used for nucleic acid detection of microorganisms in food and environment by using a fluorescent microplate reader and 3D-printed microfluidic platform. NASBA-based LOC devices have been successfully used to detect *E. coli* and Rotavirus in water and *Salmonella* in pork, beef and milk (Zhao et al., 2012; Zhai et al., 2019; Pilevar et al., 2021).

## Sample Detection in LOC for Nucleic Acid Detection of Food and Environmental Microorganisms

Besides pathogen capture, cell lysis, nucleic acid extraction, and nucleic acid amplification, LOC equipment also need a variety of sample testing methods to detect microorganisms qualitatively or quantitatively. Biosensor is a device that measures biological or chemical reactions by producing a signal proportional to the concentration of an analyte. Molecules that specifically recognize the analyte are called biological receptors. The signal generation resulting from the interaction between the biological receptors and the analyte is called biometrics, and mainly in the form of light, heat, pH, charge or mass changes. Most transducers produce optical, electrical, or other measurable signals. These energy signals are then processed and monitored by electronic equipment (Bhalla et al., 2016). Several common sensors such as fluorescence sensor, SPR sensor, SERS sensor and electrochemical biosensor are briefly introduced as below.

### Fluorescence Detection

Fluorescent probes are often used to detect nucleic acids in real-time PCR (Ahn et al., 2018; Aufdembrink et al., 2020; Salman et al., 2020; Zhou et al., 2021). Some nucleic acids have a very weak internal fluorescence that is hardly detected. Fluorescent probes are small fluorescent molecules which can enhance the fluorescence intensity after binding with nucleic acid. Fluorescence signal was then detected by fluorescence microscope and fluorescence biosensor. Fluorescent probes include organic fluorescent dyes, metal complexes, metal particles, nanoparticles, etc. (Kricka and Fortina, 2009). For example, 4′, 6-diamidino-2-phenylindole (DAPI) is a DNA-specific probe that forms fluorescent complexes by attaching to A-T-rich DNA sequences (Kapuscinski, 1995). Wei et al. (2020) designed and synthesized a small-molecule fluorescent probe based on adenine-coumarin derivative. The probe showed significant fluorescence enhancement to nucleic acid at 495 nm (DNA) and 487 nm (RNA), and the fluorescence intensity showed a good linear relationship with the concentration of nucleic acid. Peptide nucleic acids (PNAs) are synthetic DNA analogizes that can be used as probes to strongly hybridize with DNA (Park et al., 2019). Klimkowski et al. (2019) synthesized a series of thiazole orange (TO) functionalized oligonucleotides for nucleic acid detection. They found that 2′ -OME RNA probes with TO at uracil 5 or ribose 2′ were extremely effective, showing up to 44-fold fluorescence enhancement to DNA and RNA.

Nucleic acid detection based on CRISPR/Cas system also utilizes fluorescence detection methods. The CRISPR/Cas system is an adaptive immune system which is widely present in bacteria and archaea. It includes three stages: adaptation, expression, and interference. In the adaptation phase, bacteria or archaea carrying one or more CRISPR loci can integrate short DNA fragments homologous to foreign virus or plasmid sequences into host chromosomes. In the expression stage, pre-CrRNA, a primary transcript of the CRISPR gene sequence, is produced and processed into crRNA. crRNA matches the target sequence of the virus or plasmid. In the interference stage, crRNA guides Cas protein to the target sequence of virus or plasmid to form the effector complex, and uses Cas protein to cut the target sequence (Makarova et al., 2011). Nucleic acid detection based on CRISPR/Cas system mainly utilizes Cas9, Cas12, and Cas13. Cas9 has two domains, HNH domain and RuvC_like domain, which cleave complementary and non-complementary strands of target DNA, respectively. Zhang et al. (2017) mutated these two domains of Cas9 and obtained nuclease-deactivated Cas9 (dCas9). The luciferase was divided into two parts (NFluc or CFluc) and fused with two dCas9 proteins, respectively. Using two types of single guide RNA (SgRNA) complementary to the upstream and downstream segments of the target DNA sequence, two dCas9 were guided to the upstream and downstream segments of the target DNA, and the distance between the two parts of luciferase was shortened. Its catalytic activity was restored to emit light to achieve the detection effect. Different from Cas9, Cas12 and Cas13 have additional accessory cutting activity. When Cas protein forms an efficacious complex with sgRNA and target sequence, its accessory cutting activity is activated to indiscriminately cut the surrounding non-target nucleic acid that has been labeled by fluorescence, thus releasing signals for detection (Abudayyeh et al., 2016). Gootenberg et al. (2017) designed a platform called SHERLOC that utilizes this accessory cutting activity of CRISPR/Cas13a to detect Zika virus and Dengue virus. Chen et al. (2018) designed a platform called DETECTR that utilizes this accessory cutting activity of CRISPR/Cas12a to detect human papillomavirus. However, the CRISPR-based nucleic acid detection methods mentioned above still rely on traditional PCR or isothermal amplification technology to amplify target molecules. For example, SHERLOCK and DETECTR both use RPA to amplify target nucleic acids. Nucleic acid detection technology based on CRISPR/Cas system is rarely used in LOC of nucleic acid detection of food and environmental microorganisms, though it has great research prospects.

### Surface Plasmon Resonance

**FIGURE 7:**
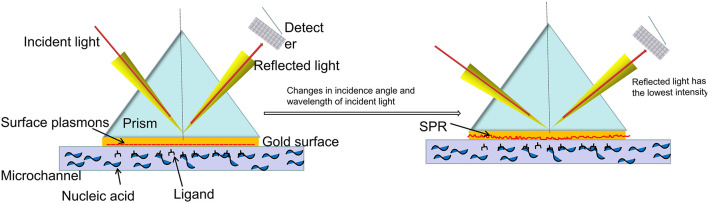
Schematic diagram of surface plasmon resonance.

The principle of Surface plasmon resonance (SPR) is shown in Figure 7. SPR is an optical phenomenon. The evanescent wave, which occurs when light is completely reflected at the glass interface, can trigger free electrons on the metal surface to produce plasmon. Under certain circumstances, the surface plasma and the evanescent wave will resonate if their frequency and wave number of are equal. Then the incident light is absorbed and the reflected light energy drops sharply, so a resonance peak will appear on the reflected spectrum when the reflected intensity reaches the lowest value. The resonance angle or resonance wavelength varies with the surface medium of metal film. Therefore, SPR spectrum can reflect changes of the surface of the metal film. The resonance angle or resonance wavelength will be changed by combining the biological receptors with the analyte (Fathi et al., 2019). Huang et al. (2020) designed a portable multi-angle scan SPR sensor that uses a motor to rotate and drive the belt to control the angle of the motor’s incident and reflected light for real-time monitoring. The nucleic acid hybridization experiment on gold film chip can obtain the sample information by observing the reflection spectrum.

### Surface-Enhanced Raman Scattering

Raman spectra is a kind of scattering spectrum, which can obtain molecular structure information by analyzing the scattering spectrum caused by different incident light frequencies. After the analyte is fixed on the metal surface through interaction with biological receptors, its molecular structure can be identified by Raman spectra. It is found that the intensity of Raman spectra can be greatly improved when the sample is adsorbed on nano-metal particles or metal pieces with rough surface, which is called surface enhanced Raman scattering (SERS) (Butler et al., 2016; Barbosa et al., 2021). Yu H. et al. (2020) developed a new SERS-based sensor, which uses gold nanoflowers (AuNFs) to improve the intensity of Raman spectra. The sensor enables sensitive and quantitative analysis of biomolecules. It can distinguish different bacteria with a sensitivity as low as a single bacteria, suggesting a great application prospect. Zhou et al. (2020) designed an SERS-based biosensor for the detection of *E. coli O157:H7* from food. Aptamer (APT-1) and signaling molecule Rhodamine B (RhB) were bound to gold nanorods (GNRs) to form a gold nanobone (NBs). Apt-1 and RhB were embedded in NBs, and the combination showed good recognition, excellent stability and significant enhancement of Raman signal strength in the detection of *E. coli O157: H7*.

### Electrochemical Biosensors

Biological reactions often cause consumption or production of electrons or ions, and the reaction between the receptors and the targets is no exception. The electrical properties of the solution, such as potential and current, may thus change. The transducer can be used to transform the biological signal into a detectable electrical signal proportional to the target concentration (Ferapontova, 2018; Wu et al., 2019). Conductance/impedance, amperometric/voltammetric, and potentiometric methods are the most commonly used electrochemical biosensing methods that can be integrated into microfluidic devices (Kant et al., 2018). Park et al. (2018a) designed a membrane based integrated chip to detect *Staphylococcus aureus* and *E. coli* in food. The system can simultaneously perform nucleic acid amplification and electrochemical detection, and accurately analyze the target pathogen genes by square wave voltammetry (SWV) in 25 s. But before these samples can be used in the developed sensor, DNA must be extracted and purified, which can take a long time. Liébana et al. (2016) proposed an electrochemical magneto-genosensing approach for the detection of *Salmonella*, *Listeria*, and *E. coli.* This method used a set of specific primers for each pathogen, followed by electrochemical magneto-genosensing on silica magnetic particles. Park et al. (2018b) produced highly ordered nanocolumnar electrodes by means of soft lithography and metal evaporation. It had wide electrochemical and mechanical properties and wide reaction space, which could be used for sensitive analysis. The gold and silver electrodes prepared on the nanocolumn array shows strong and stable electrochemical performance and can detect amplified genes from foodborne *E. coli* pathogens.

## Summary and Prospect

Microfluidic technology integrates sample preparation, nucleic acid amplification, and sample detection on a chip. The technology of nucleic acid amplification and sample detection has been well developed, and a variety of emerging experimental technologies have been timely designed and implemented on chip. However, the development of sample preparation technology is relatively slow, and most LOC equipment still needs sample pretreatment in advance, which is inconsistent with the concept of micro total analysis system and LOC. As mentioned above, the microfluidic chip integrated with real-time fluorescence loop-mediated isothermal amplification technology developed by Zhou et al. (2021) has good sensitivity and specificity, but preprocessing of samples is required to enrich target DNA. The integrated chip designed by Park et al. (2018a) for the detection of *Staphylococcus aureus* and *E*. *coli* in food integrates nucleic acid amplification and electrochemical detection, but also requires advance DNA extraction and purification. This makes them unable to meet the requirements of POCT. In fact, most LOC equipment used for food and environmental microbiological detection have not yet been integrated with sample processing equipment. Although several techniques have been developed for sample preparation in LOC, including microsphere, filter or membrane, dielectrophoresis, magnetophoresis, acoustophoresis, an equipment that can directly test the obtained samples without sample processing is the ideal LOC. For example, Sun et al. (2015) reported on an 8-chamber LOC system that integrates microsphere-based sample preparation, LAMP, and real-time fluorescence detection for rapid quantitative detection of *Salmonella* in food samples. The entire diagnostic procedure is performed in a single chamber, and up to eight samples can be processed simultaneously. Yin et al. (2020) successfully detected *E*. *coli O157:H7, Listeria* monocytogenes and *Salmonella enteritidis* in milk within 45 min by integrating magnetic bead enrichment target nucleic acid and multiple digital RPA (ImdRPA) into microfluidic chip. Chen et al. (2015) used magnetic nanoparticles to extract genomic DNA from lytic bacteria and used HDA amplification to detect *Staphylococcus aureus* in dairy and meat products. The detection limit was 5 × 10^0^ CFU/mL for milk powder samples and 5 × 10^1^ CFU/mL for pork samples in less than 2 h. The detection time would be greatly reduced if the sample preparation can be integrated into the LOC device (Table 2).

**TABLE 2:** Five typical Lab-on-chip (LOC) devices for detection of microbial nucleic acid in food and environment.

Pathogen	Sample	Nucleic acid extraction method	Nucleic acid amplification method	Sample test method	Detection limit	Detection time	References
*Salmonella*	Pork	Immunomagnetic beads	LAMP	Real-time fluorescence detection.	50 cells per test.	40 min	Sun et al., 2015
*E. coli O157: H7, Listeria monocytogenes and Salmonella*	MILK	Magnetic bead	RPA	Real-time fluorescence detection.	10 cells for each kind of pathogen.	15 min	Yin et al., 2020
*Staphylococcus aureus*	Milk powder and pork	Silica-coated magnetic nanoparticles	HDA	Fluorescence detection	5 × 10 (1) CFU/mL for milk powder samples and 5 × 10 (1) CFU/mL for pork samples.	2 h	Chen et al., 2015
*Staphylococcus aureus*	Air	Don’t require.	LAMP	Fluorescence detection	27 cells per test.	4 h and 40 min	Jiang et al., 2016
*E. coli O157: H7*	Orange juice and milk.	Poly-aptamers modified microchannels	RCA	Fluorescence detection	80 cells/mL	—	Jiang et al., 2020

Another challenge for LOC development is interaction between biomolecules and wall of the microfluidic channel. The extremely high surface-to-volume ratio of microchannels may lead to a high incidence of non-specific adsorption and surface effects, that may limit or inhibit amplification reactions. Proper surface treatments, such as polymer coatings with polyethylene glycol (PEG) and linear polyacrylamide (LPA), or sealing with bovine serum albumin, are required to mitigate these effects (Asiello and Baeumner, 2011).

At present, microfluidics and LOC technologies are booming. With the help of scientists in biology, medicine, chemistry, electronics, materials and other fields, we believe that LOC for nucleic acid detection of food and environmental microorganisms will finally meet the “ASSURED” principles as proposed by WHO, and play an important role in the real-time detection of pathogenic microorganisms in food and environment.

## Author Contributions

YX and ML contributed to study concept and design. LY, WY, FS, MX, ZZ, XB, and JD collected and sorted out literatures. LY, YX, and ML drew pictures. LY, WY, and FS wrote the first draft. YX and ML edited the English version. JD, YX, and ML approved the submitted version after modification. All authors contributed to the article and approved the submitted version.

## Conflict of Interest

The authors declare that the research was conducted in the absence of any commercial or financial relationships that could be construed as a potential conflict of interest.

## Publisher’s Note

All claims expressed in this article are solely those of the authors and do not necessarily represent those of their affiliated organizations, or those of the publisher, the editors and the reviewers. Any product that may be evaluated in this article, or claim that may be made by its manufacturer, is not guaranteed or endorsed by the publisher.
